# Mitotic entry: Non-genetic heterogeneity exposes the requirement for Plk1

**DOI:** 10.18632/oncotarget.5507

**Published:** 2015-10-13

**Authors:** Claire F. Aspinall, Daniella Zheleva, Anthony Tighe, Stephen S. Taylor

**Affiliations:** ^1^ Faculty of Life Sciences, University of Manchester, Manchester M13 9PT, United Kingdom; ^2^ Cyclacel Ltd, Dundee DD1 5JJ, United Kingdom

**Keywords:** mitosis, Plk1

## Abstract

The quest to develop novel antimitotic chemotherapy agents has led to the generation of several small molecule inhibitors targeting Plk1, a protein kinase required for multiple aspects of cell division. Previous studies have shown that upon exposure to Plk1 inhibitors, cells enter mitosis, delay briefly in prophase and then arrest in mitosis due to an inability to undergo centrosome separation. Here, we show that four different classes of Plk1 inhibitor block mitotic entry in several cancer cell lines and non-transformed RPE-1 cells. The proportion of cells that arrest in G2 is cell line and concentration dependent, and is subject to non-genetic heterogeneity. Following inhibitor washout, the G2 block is alleviated and cells enter mitosis but then fail to complete cell division indicating that most Plk1 inhibitors are not fully reversible. An exception is CYC140844; in contrast to five other inhibitors examined here, this novel Plk1 inhibitor is fully reversible. We discuss the implications for developing Plk1 inhibitors as chemotherapy agents and research tools.

## INTRODUCTION

Antimitotic agents, including the taxanes, are used extensively to treat breast and ovarian cancer as well as various leukemias [1]. All clinically relevant antimitotic agents inhibit microtubule dynamics, and while this has potential to impact multiple aspects of tumor biology, microtubule function is particularly important during cell division when the mitotic spindle apparatus is assembled [2, 3]. In cell culture and breast cancers, disrupting spindle function causes mitotic failure, in turn reducing proliferative potential [1, 4, 5]. However, patient responses to antimitotic agents are unpredictable, resistance is common, and toxicity in the form of neuropathies can be problematic [6–8]. To address these limitations, a plethora of second generation antimitotic agents have been developed, including excellent drugs targeting mitotic kinesins, such as Eg5/KSP and Cenp-E, and mitotic kinases, such as Plk1, Aurora A and Aurora B [9–11].

Plk1, a member of the polo-like kinase family, regulates multiple cell cycle processes, including DNA replication, recovery from G2 checkpoint arrest, entry into mitosis, centrosome maturation, bipolar spindle formation, kinetochore-microtubule attachment, activation of the anaphase promoting complex/cyclosome, resolution of sister chromatid cohesion, and cytokinesis [12–17]. Plk1 is overexpressed in a variety of human tumors and is often an indicator of poor patient prognosis [11, 18]. Consistent with a pro-tumorigenic role, overexpressing Plk1 activates FoxM1, stimulating transcription of several mitotic regulators favoring cell proliferation [19]. Moreover, constitutive expression of Plk1 transforms NIH 3T3 fibroblasts [20], while inhibiting Plk1 induces cell-cycle arrest and apoptosis [21, 22].

To explore Plk1's potential as an anti-cancer drug target, several small molecule inhibitors have been developed and a number are undergoing clinical evaluation [23–25]. However, while our understanding of Plk1 biology is well advanced, the long-term fate of cells exposed to Plk1 inhibitors is less clear. Therefore, we set out to examine cell fate in response to Plk1 inhibitors using a single-cell-based time-lapse microscopy approach that previously revealed extensive intra- and interline variation when cancer cells are exposed to microtubule toxins [26].

## RESULTS

### 100 nM BI 2536 induces a penetrant polo phenotype

**Figure 1:**
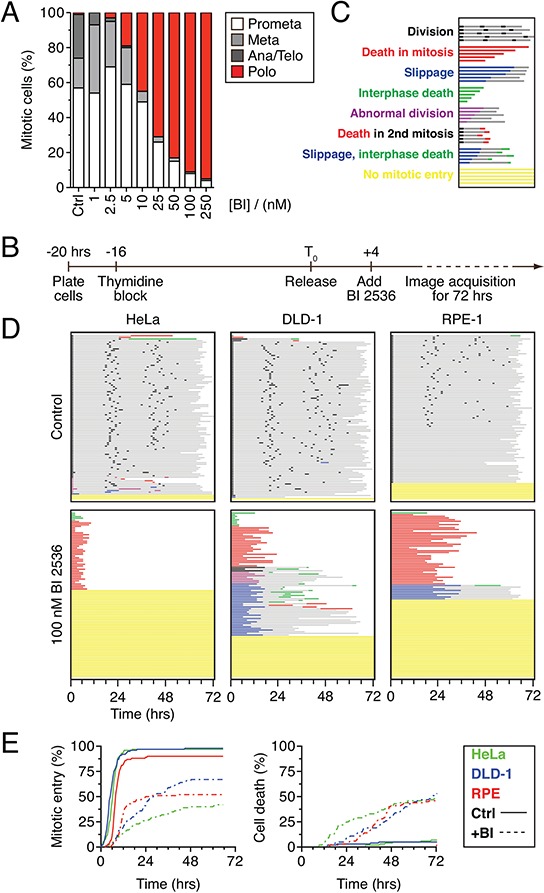
BI 2536 blocks mitotic entry **A.** Asynchronous HeLa cells were treated for two hours with BI 2536 at the concentrations indicated, fixed and stained for the mitosis marker phospho-histone H3 (Ser10) and α-tubulin. Mitotic cells that had undergone nuclear envelope breakdown were then assigned to one of four stages based on spindle morphology. 100 cells were scored for each concentration. **B.** Timeline of time-lapse imaging strategy. **C.** Key to cell fate profiles. Each individual cell is represented by a horizontal line. Color and length of the line indicate the behavior of the cell and the time elapsed respectively. **D.** Fate profiles of HeLa, DLD1 and RPE-1 cell lines expressing GFP-H2B exposed to 100 nM BI 2536 for 72 hours. 100 cells were analyzed per condition. **E.** Analysis of data collected in (D) Left panel: cumulative mitotic entry frequency; only the first mitosis is shown. Right panel: cumulative cell death frequency, including apoptosis in mitosis and death in interphase.

When asynchronous populations of various cell lines are exposed to Plk1 inhibitors, cells undergo mitotic arrest exhibiting the prototypical “polo” phenotype that arises when centrosomes fail to separate [27–30]. To define the long-term fate of these cells, we first focused on the pioneer Plk1 inhibitor, BI 2536 [27]. To determine the minimal drug concentration required to induce a penetrant polo phenotype, HeLa cells were exposed to increasing concentrations of BI 2536 for two hours and spindle morphology analyzed by immunofluorescence microscopy (Fig. 1A). At concentrations of 5 nM and below, cells were capable of assembling bipolar, metaphase spindles. However, at 10 nM and above, metaphase figures were rare and polo spindles became increasingly prevalent. At 100 nM, more than 90% of mitotic cells were classified as polo, so this concentration was chosen as a starting point for further experiments.

### BI 2536 blocks mitotic entry in transformed and non-transformed cells

To define the long-term fate of Plk1 inhibition, HeLa cells expressing a GFP-tagged histone H2B were synchronized using a single thymidine block, released for four hours, then exposed to 100 nM BI 2536 (Fig. 1B). One hour later, the cells were subjected to fluorescence time-lapse microscopy for 72 hours. Image sequences were then analyzed manually; 100 randomly selected cells were tracked and chromatin morphology used to monitor mitotic progression and apoptosis. To represent the data in a manner that facilitates comparative analyses without obscuring the complexity, we plotted fate profiles as described previously ([26] and see Fig. 1C). Note that unless stated otherwise, zero hours represents when a cell entered mitosis. In the absence of BI 2536, 70% of the cells underwent three cell divisions during the 72 hour period, indicating that the imaging conditions were relatively benign (Fig. 1D). In the presence of BI 2536 a substantial proportion of cells underwent mitotic arrest with a polo phenotype, followed several hours later by apoptosis (Fig. 1D, red lines), consistent with previous observations [27]. However, only 43% of cells entered mitosis, arrested and died; 52% of the cells never entered mitosis during the 72 hour period (Fig. 1D, yellow lines). To determine if other cell lines behave similarly, we analyzed DLD-1 cells, a diploid, chromosomally stable colon cancer line, and hTERT-immortalized non-transformed RPE-1 cells. DLD-1 cells are slippage prone [26], i.e. following a prolonged mitotic arrest, they tend to exit mitosis and return to interphase without completing cell division [31]. Accordingly, while 25% of the BI 2536-treated DLD-1 cells arrested then died, 32% underwent slippage (Fig. 1D, blue lines). As observed with HeLa, 30% of the DLD-1 cells failed to enter mitosis. The mitotic behavior of the RPE-1 cells was similar to the cancer cells; 43% arrested then died while 9% slipped, albeit after much longer arrest periods (25.5 hours on average vs. 5.5 hours and 12.0 hours in HeLa and DLD-1 respectively). Once again however, a substantial proportion, 47%, never entered mitosis, remaining in interphase for the duration of the experiment (Fig. 1D). Thus, as we showed previously with taxol, nocodazole and the Eg5 inhibitor AZ138 [26], the Plk1 inhibitor BI 2536 gives rise to intra- and interline variation, with cells either undergoing death in mitosis or slippage following a prolonged mitotic arrest. However, in contrast to the aforementioned drugs, BI 2536 also blocks mitotic entry in a substantial proportion of cells.

### When cells commit to mitosis in the presence of BI 2536, entry is delayed

Previously, BI 2536 was shown to induce a brief prophase delay [27]. We therefore asked whether cells that did commit to mitosis experienced a delay by plotting the cumulative mitotic entry frequency (Fig. 1E). Curves derived from the control populations rose sharply and plateaued just short of 100%, demonstrating that the vast majority of cells entered mitosis within 12 hours. Consistent with a substantial proportion of cells failing to enter mitosis, the curves derived from drug-treated populations plateaued well below 100%. However, the curves were also substantially shallower, indicating that when cells did commit to mitosis, entry was delayed. Consistent with mitotic entry being required for antimitotic induced apoptosis, the cumulative cell death frequency curves also plateaued well below 100% (Fig. 1E). Thus, these observations confirm that when cells enter mitosis in the presence of BI 2536, entry is delayed and that following mitotic entry, subsequent cell cycle progression is blocked leading to cell death. However, because a substantial proportion of BI 2536-treated cells fail to enter mitosis, they are protected from the apoptotic response that is typically induced by antimitotic agents.

### BI 2536 induces a penetrant mitotic entry block in RKO cells

**Figure 2:**
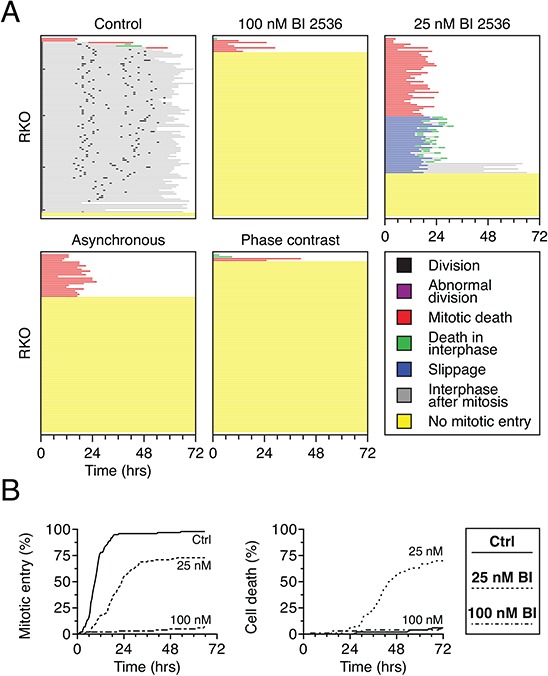
BI 2536 blocks mitotic entry in RKO cells **A.** Cell fate profiles of GFP-H2B RKO cells, either thymidine-synchronized or asynchronous, exposed to either 25 nM or 100 nM BI 2536 for 72 hours. Image capture was performed either by fluorescence or phase contrast microscopy. 100 cells were analyzed per condition. **B.** Analysis of data collected in (A) Left panel: cumulative mitotic entry frequency; only the first mitosis is shown. Right panel: cumulative cell death frequency, including apoptosis in mitosis and death in interphase.

While testing the effect of BI 2536 on various cell lines, we noticed that mitotic entry was profoundly affected in RKO cells, with more than 90% of the population failing to enter during the 72-hour imaging period (Fig. 2A). We therefore chose RKO cells as a model system for further analysis. To determine whether the mitotic entry failure was a synthetic effect due to the combination of the thymidine-based synchronization and Plk1 inhibition, we repeated the experiment using an asynchronous population, i.e. without the thymidine block and release. Under these conditions, the vast majority of cells still failed to enter mitosis (Fig. 2A). Similarly, to determine whether the fluorescent imaging procedure was inducing a synthetic effect, we used phase-contrast imaging to follow cell fate. When synchronized RKO cells were exposed to 100 nM BI 2536 and analyzed by phase-contrast imaging, greater than 95% of cells failed to enter mitosis (Fig. 2A). Thus, the failure to enter mitosis does not appear to be a consequence of damage induced by either the thymidine synchronization or the fluorescent imaging. Interestingly, 24% of cells in the asynchronous population entered mitosis, indicating that RKO cells are capable of entering mitosis in the presence of BI 2536. To explore this further, we reduced the drug concentration to 25 nM. Under these conditions, 76% of cells entered mitosis and arrested, with 44% undergoing death in mitosis and 26% undergoing slippage followed by post-mitotic apoptosis (Fig. 2A, green lines). This suggests that while penetrant Plk1 inhibition blocks mitotic entry in RKO cells, partial inhibition allows mitotic entry but then disrupts spindle assembly to an extent sufficient to trigger mitotic arrest. Analysis of the cumulative mitotic entry frequencies confirmed this (Fig. 2B). At 100 nM BI 2536, mitotic entry was almost completely blocked, while at 25 nM, mitotic entry was delayed but approached 75%. These observations raise an interesting paradox, highlighted by the cumulative death frequencies (Fig. 2B). Specifically, the higher concentration of BI 2536 protected cells from apoptosis by blocking mitotic entry. By contrast, the lower drug concentration permitted mitotic entry thus leading to extensive apoptosis. These results reveal that different levels of Plk1 activity are required for different aspects of cell division; specifically more activity is required to assemble a bipolar spindle than is required to enter mitosis.

### BI 2536 blocks cells in late G2

**Figure 3:**
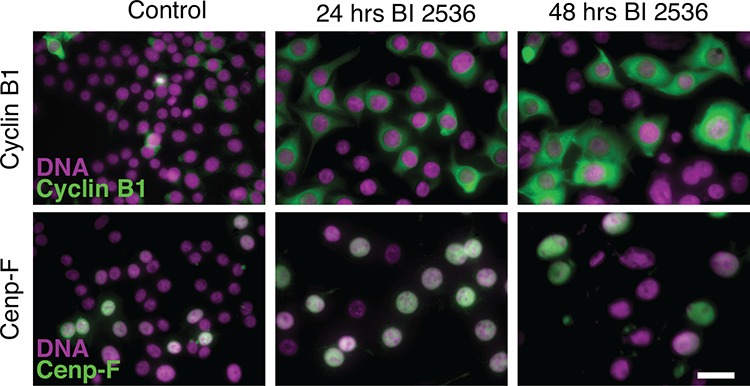
BI 2536 blocks cells in late G2 Asynchronous RKO cells were treated for 24 or 48 hours with 100 nM BI 2536, fixed and stained to detect Cyclin B1 (top panel), Cenp-F (bottom panel) and the DNA. Scale bar 10 μm.

In light of Plk1's known ability to regulate progression into mitosis, we reasoned that the cell cycle block induced by BI 2536 was likely to be in late G2. To test this we analyzed two G2 markers, namely Cenp-F and Cyclin B1. Cenp-F levels rise in S-phase, accumulating in the nucleus, reaching a maximum in G2 before degradation during late mitosis [32, 33]. Cyclin B1 increases in late S-phase, accumulating in the cytoplasm, then translocates into the nucleus at the onset of mitosis, followed by degradation at anaphase [34]. RKO cells were exposed to 100 nM BI 2536 for 24–48 hours, then fixed and stained to detect Cenp-F, Cyclin B1 and the DNA. Consistent with the BI 2536 inducing a G2 arrest, at the 24 hour time point, 80% of the nuclei were relatively large and stained positive for Cenp-F, while the cytoplasm stained strongly for Cyclin B1 (Fig. 3). By 48 hours, cells positive for Cenp-F and Cyclin B1 dropped to approximately 60% and at later time points the nuclei seemed unusually large (not shown). One possibility is that following a prolonged G2 delay, the cells skipped mitosis altogether, returning to interphase and thereby triggering degradation of Cenp-F and Cyclin B1. A similar phenomenon has been described following Cdk1 inactivation in HT1080 cells [35]. Mitotic skipping has also been observed in MCF7 cells treated with ionizing radiation [36]. While further analysis will be required to confirm whether or not BI 2536-treated cells can skip mitosis, the simplest explanation for our observations is that at the 24 hour time point, the cells are indeed arresting in G2.

### Mitotic entry in the presence of BI 2536 is subject to non-genetic heterogeneity

**Figure 4:**
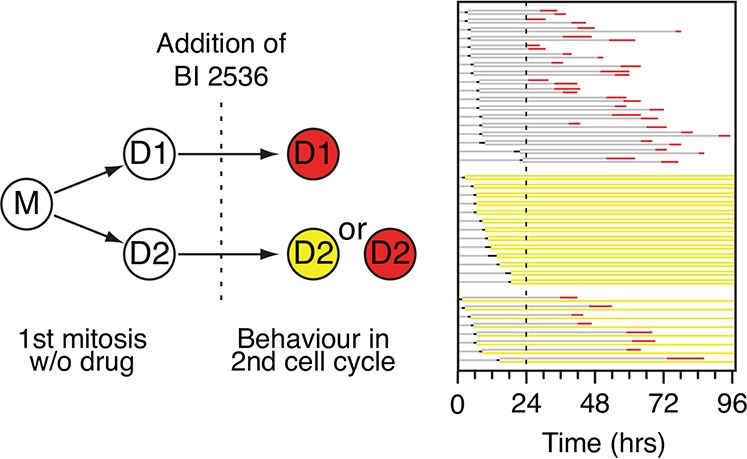
Analysis of daughter pairs Experimental strategy and fate profile of GFP-H2B HeLa cells. Mother cells (M) were followed through one mitosis in the absence of drug to identify daughter cells (D1 and D2). BI 2536 was then added and the cells followed for 72 hours. 39 cells were analyzed and grouped according to daughter cell fate.

Within a population of HeLa cells, approximately half entered mitosis in the presence of BI 2536, while the other half arrested in G2 (Fig. 1D). While this difference might be explained by genetic variation in the population, we previously showed that cell fate following prolonged mitotic arrest is subject to non-genetic heterogeneity [26]. Specifically, when we followed pairs of daughter cells exposed to an Eg5 inhibitor, their fate was often different; while one sister underwent death in mitosis, the other underwent slippage. Because genetic drift between newly formed daughters is expected to be minimal, these different behaviors are better explained by non-genetic heterogeneity. We therefore asked whether the ability to enter mitosis in the presence of BI 2536 was subject to non-genetic heterogeneity. Asynchronous, untreated GFP-H2B HeLa cells were analyzed by time-lapse microscopy to identify pairs of the daughter cells (Fig. 4). 100 nM BI 2536 was then added and imaging continued for a further 72 hours. Daughter pairs were tracked to determine whether they entered mitosis or arrested in G2. Of 39 pairs, in 18 cases both daughters entered mitosis, arrested then died (Fig. 4). In 13 cases, both daughter cells arrested in interphase. However, in eight cases, one daughter entered mitosis and died while the other never entered mitosis. Thus overall, approximately half the population underwent each fate, consistent with the data in Fig. 1D. However, while 79% of the daughter pairs underwent the same fate, 21% exhibited different responses. This different behavior could be explained by stochastic genetic variation, e.g. arising via chromosome missegregation, but a more likely explanation is that stochastic variation in the stability of the networks regulating entry into mitosis renders cells differentially sensitive to Plk1 inhibition. Consistent with this notion, the duration of G2 arrest following DNA damage prior to Plk1-dependent recovery is also subject to non-genetic heterogeneity [37].

### Washout of BI 2536 releases the G2 block

**Figure 5:**
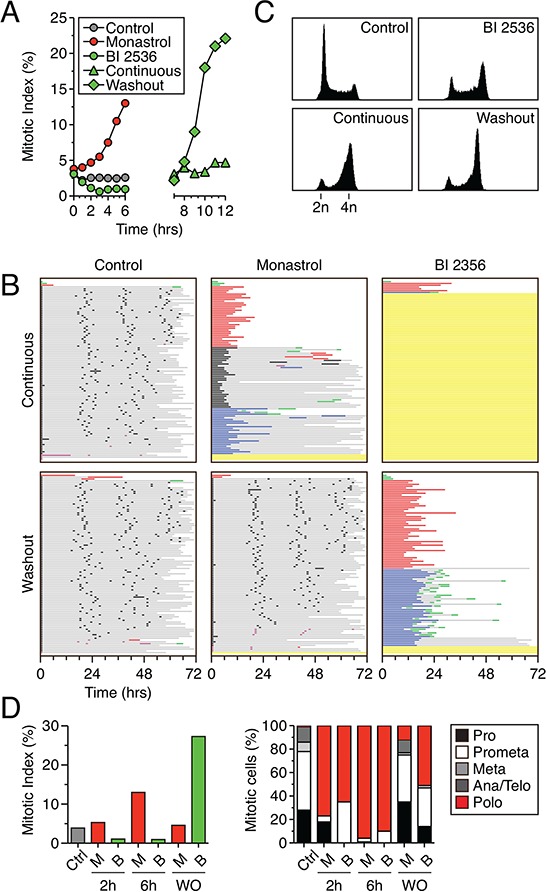
RKO cells arrest in mitosis following washout of BI 2536 **A.** Mitotic index as determined by phospho-histone H3 (Ser10) staining of RKO cells treated with either 100 μM monastrol or 100 nM BI 2536. Cells were either exposed continually or the BI 2536 was washed out after 6 hours. **B.** Cell fate profiles of RKO cells exposed to either 100 μM monastrol or 100 nM BI 2536, either continuously for 72 hours or after washout following a six hour exposure. 100 cells were analyzed per condition. **C.** DNA content profiles shown for control or 100 nM BI 2536 treated cells after 6 hours (top row) or after 12 hours (bottom row) of either continuous exposure or after inhibitor washout. **D.** Asynchronous RKO cells were treated with either 100 nM BI 2536 (B) or 100 μM monastrol (M), fixed and stained for phospho-histone H3 (Ser10) and α-tubulin. Left panel; mitotic index as determined by percentage of cells staining positive for phospho-histone H3 (Ser10). Right panel; mitotic stage and spindle morphology as determined visually using α-tubulin staining.

Following DNA damage, Plk1 activity is required to re-start the cell cycle and drive entry into mitosis [14, 38]. We therefore set out to determine whether restoring Plk1 activity in BI 2536-treated cells would re-start the cell cycle following a prolonged G2 arrest. To address this we asked whether the G2 arrest induced by BI 2536 is reversible. Asynchronous RKO cells were treated with 100 nM BI 2536 for six hours then the drug washed out and replaced with fresh media. At hourly time points, the cells were fixed and stained to detect histone H3 phosphorylated on serine 10 to determine the mitotic index. As an additional control, we analyzed cells treated with the Eg5 inhibitor monastrol, which like BI 2536 prevents centrosome separation and thus induces a mitotic arrest, but unlike BI 2536 does not influence mitotic entry [39]. In the untreated population, the mitotic index remained constant at approximately 2.5% (Fig. 5A). In the monastrol-treated culture, the mitotic index rose to ~13% within 6 hours, consistent with mitotic entry followed by cell cycle arrest. Consistent with blocking mitotic entry, the mitotic index of the BI 2536-treated population fell to less than 1%. Strikingly, following washout of BI 2536, the mitotic index rose sharply, climbing to ~22% within 6 hours. Thus, the G2 block induced by BI 2536 can be alleviated by drug washout.

### Following washout of BI 2536, cells arrest in mitosis

To determine whether RKO cells could complete mitosis following washout of BI 2536, we performed cell fate profiling. In the absence of drugs, the vast majority of RKO cells underwent three cell divisions during a 72-hour imaging period (Fig. 5B). When continuously exposed to monastrol, virtually all the cells arrested in mitosis; 34% eventually divided, 34% died in mitosis and 26% underwent slippage. However, following washout after a six hour monastrol exposure, the vast majority of cells underwent three cell divisions, demonstrating that cells can in principle fully recover. When continuously exposed to BI 2536, the vast majority of RKO cells arrested in G2, consistent with the data in Fig. 2A. When BI 2536 was washed out after a six hour exposure, virtually all the cells entered mitosis (Fig. 5B), consistent with the data in Fig. 5A. Strikingly however, these cells did not complete mitosis; 93% of the population arrested in mitosis for an average of 17 hours, with 49% then dying in mitosis and 43% undergoing slippage. Of those that slipped, the vast majority then died in the subsequent interphase. Flow cytometry analysis confirmed this: twelve hours following washout after a six hour BI 2536 exposure, most cells had a 4n DNA content, consistent with a failure to complete cell division (Fig. 5C). In line with Plk1's known function, we reasoned that the mitotic arrest following washout of BI 2536 was most likely due to the inability to assemble a bipolar spindle. To test this we analyzed spindle morphology following washout of BI 2536. RKO cells were treated with 100 nM BI 2536 for six hours, the drug washed out and the cells then fixed six hours later. Consistent with the data in Fig. 5A, the mitotic index increased following washout (Fig. 5D). Moreover, 50% of these cells displayed the polo phenotype and metaphases and anaphases were rare (Fig. 5D). By contrast, following a monastrol washout, monopolar figures were rare and metaphases and anaphases were readily apparent (Fig. 5D). Thus, the immunofluorescence analysis, the flow cytometry profiles and the time-lapse imaging all paint a consistent picture; following washout of BI 2536, the G2 block is alleviated and cells enter mitosis, but cell division is blocked due to the inability to assemble a functional spindle.

### Two possibilities could explain incomplete recovery following washout of BI 2536

To explain why RKO cells failed to complete division following washout of BI 2536, we considered two possibilities. The first was that the drug simply failed to washout completely so that Plk1 activity was not fully restored; note that the phenotype following washout of 100 nM BI 2536 (Fig. 5B) is not dissimilar to that observed in the continuous presence of 25 nM (Fig. 2A). The second possibility is that following washout, Plk1 activity is fully restored but the cell cycle has passed a potential execution point where Plk1 activity primes the cell for spindle assembly. To distinguish between these two possibilities, we set out to analyze other Plk1 inhibitors, reasoning that if the second explanation was correct then the inability to fully recover from washout should be a common feature of all Plk1 inhibitors.

### Full recovery following washout of the novel Plk1 inhibitor CYC140844

**Figure 6:**
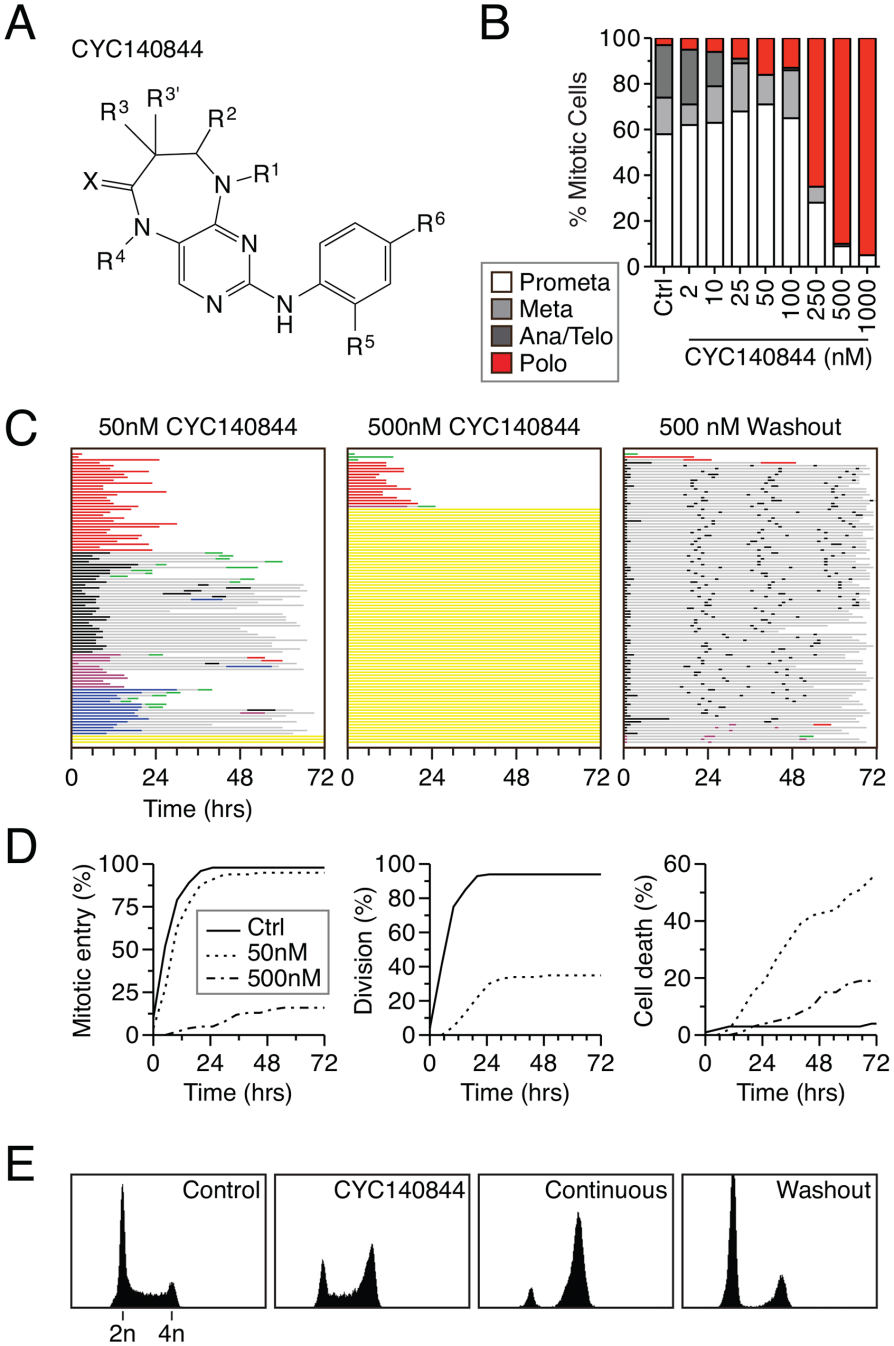
Full recovery following washout of CYC140844 **A.** Chemical structure of CYC140844. **B.** Asynchronous HeLa cells were treated for two hours with CYC140844 at the concentrations indicated, fixed and stained for the mitosis marker phosphor-histone H3(Ser10) and α-tubulin. Mitotic cells that had undergone nuclear envelope breakdown were then assigned to one of four stages based on spindle morphology. 100 cells were scored for each concentration. **C.** Fate profiles for RKO H2B-GFP cells exposed to either 50 or 500 nM CYC140844 continually for 72 hours or after washout. 100 cells were analyzed per condition. **D.** Analysis of data collected in (C) Left panel: cumulative mitotic entry frequency; only the first mitosis is shown. Middle Panel: cumulative successful cell division frequency; only the first successful division is shown. Right Panel: cumulative cell death frequency, scoring death occurring at all stages of the cell cycle. **E.** DNA content profiles shown for control or CYC140844 treated cells after 6 hours, or after 12 hours of either continuous exposure or after washout.

CYC140844, a pyrimidodiazepinone, is a novel, selective Plk1 inhibitor (Fig. 6A, S1 and see ref. [40]). In enzyme assays, CYC140844 inhibits Plk1, Plk2 and Plk3 with IC_50_ values of 36, 113 and 702 nM respectively. When used at a single concentration of 10 μM, CYC140844 did not inhibit Akt, Aurora A, Cdk1/Cyclin B1, Cdk2/Cyclin E, Cdk7/Cyclin H, Cdk9/Cyclin T1, Flt3, Lck or VEGFR1 by more than 50%. When asynchronous HeLa cells were exposed to CYC140844, the predicted polo phenotype manifested, with 500 nM required to yield a penetrant effect (Fig. 6B). Time-lapse imaging of synchronized RKO cells showed that 50 nM induced a prolonged mitotic arrest, with 35% of cells eventually dividing normally, 34% undergoing death in mitosis and 16% undergoing slippage (Fig. 6C). At 500 nM, CYC140844 potently blocked mitotic entry. Thus, as with BI 2536, apoptosis induction by CYC140844 was more efficient at the relatively lower concentration that permitted mitotic entry (Fig. 6D). Importantly, when CYC140844 was washed out following a six hour exposure, cells not only entered mitosis but also successfully completed cell division. Indeed, following washout, the vast majority of the population divided three times and 48% completed four divisions (Fig. 6C). Consistent with full recovery, flow cytometry showed that 12 hours following washout the vast majority of cells had 2n DNA content, indicating successful cell division (Fig. 6E). Thus, in contrast to BI 2536, RKO cells appear to recover fully from a transient exposure to CYC140844. This suggests therefore that the inability to fully recover following transient BI 2536 exposure is not due to an execution point issue but rather because it does not washout well enough to allow full restoration of Plk1 activity.

### Analysis of different Plk1 inhibitors

**Figure 7:**
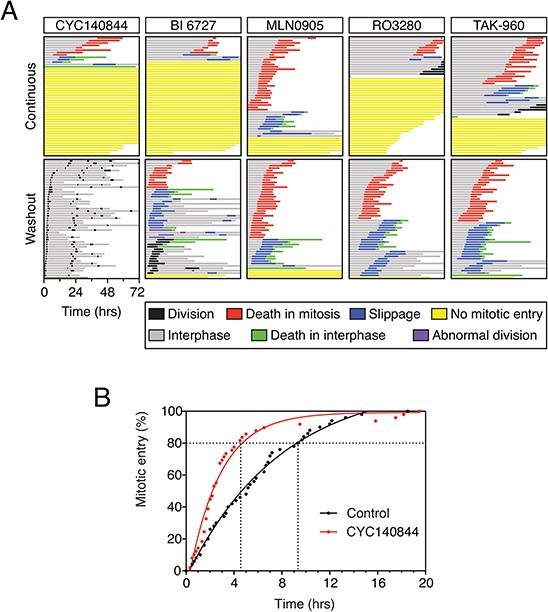
Comparison of Plk1 inhibitors **A.** Fate profiles of RKO cells exposed to 500 nM CYC140844, 500 nM BI 6727, 4 μM MLN0905, 4 μM RO3280 and 3 μM TAK-960, either continuously or after washout following an eight hour exposure. Note that zero hours represents when imaging started. **B.** Cumulative mitotic entry frequency following an eight hour exposure to 500 nM CYC140844; only the first mitosis is shown.

Because the reversibility characteristics of BI 2536 and CYC140844 are clearly rather different, we asked whether other commercially available Plk1 inhibitors behaved like BI 2536 or CYC140844. We focused on four different inhibitors, namely BI 6727, MLN0905, RO3280 and TAK-960 [30, 41–43], comparing them directly with CYC140844. RKO cells were analyzed in both the continuous presence of inhibitor and following washout after an eight hour exposure. 500 nM BI 6727 induced a penetrant interphase block, and following washout the vast majority of cells arrested in mitosis (Fig. 7A). Thus, like BI 2536, BI 6727 does not wash out well. Most cells treated with 4 μM MLN0905 entered mitosis then arrested, with 62% then dying. While only 16% of the MLN0905-treated cells failed to enter mitosis, we were reticent to increase the drug concentration further due to the possibility of off-target effects. However, following washout, the profile was very similar indicating that MLN0905 also does not wash out well. Like CYC140844 and BI 6727, 4 μM RO3280 induced a penetrant interphase block. In addition, when cells did commit to mitosis, entry was substantially delayed (Fig. 7A). Note that in the fate profiles shown in Fig. 7, zero hours represents when imaging started, allowing visualization of the time to mitotic entry. Following washout, the timing of mitotic entry was advanced but now virtually all the cells arrested in mitosis, followed by either death or slippage. 3 μM TAK-960 behaved similarly, inducing an interphase block in 32% of cells and substantially delaying mitotic entry in the rest. Upon washout, mitotic entry was advanced but then the vast majority arrested in mitosis, again followed by either death or slippage. Thus, of all the Plk1 inhibitors we analyzed, the only one compatible with full recover following washout is CYC140844.

### Combining BI 2536 and MLN8054 does not exacerbate the mitotic entry block

**Figure 8:**
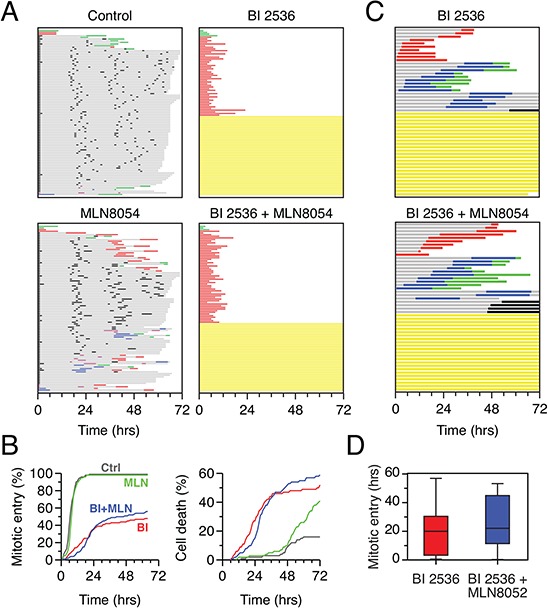
Co-inhibition of Plk1 and Aurora A **A.** Fate profiles of HeLa H2B-GFP cells exposed to 100 nM BI 2536, 1 μM MLN8054 or both for 72 hours. 100 cells were analyzed per condition. **B.** Analysis of data collected in (A) Left panel: cumulative mitotic entry frequency; only the first mitosis is shown. Right panel: cumulative cell death frequency, including apoptosis in mitosis and death in interphase. **C.** Fate profiles of DLD-1 cells exposed to 500 nM BI 2536 alone or in combination with 2 μM MLN8054. Note that zero hours represents when imaging started. **D.** Quantitation of data in (C) showing time to mitotic entry. Box-and-whisker plots show the mean, interquartile ranges and full range.

Aurora A cooperates with Plk1 to activate Cdk1 and drive mitotic entry [44–46]. Therefore, we set out to determine whether inhibition of Aurora A exacerbated the mitotic block induced by Plk1 inhibition. To do this, we returned to cell lines that exhibited a mixed response to BI 2536, namely HeLa and DLD-1 and asked whether simultaneous inhibition of both kinases increased the proportion of cells that arrested in G2. First, we treated HeLa cells with the Aurora A inhibitor MLN8054 [47], alone and in combination with BI 2536. As expected, 2 μM MLN8054 disrupted normal mitotic progression [48], but did not delay mitotic entry (Fig. 8A, 8B). Somewhat surprisingly, combining MLN8054 with BI 2536 did not increase the proportion of cells that blocked in interphase (Fig. 8A). Also, of the cells that did commit to mitosis, MLN8054 did not enhance the delay induced by BI 2536 (Fig. 8B). In DLD-1 cells, combining MLN8054 with BI 2536 also did not increase the proportion of cells blocked in interphase (Fig. 8C). However, the time to mitotic entry did appear to be lengthened (Fig. 8C, note that as in Fig. 7, for the fate profiles shown in Fig. 8C, zero hours represents when imaging started). Indeed, quantitation shows that on average, BI 2536-treated cells took 18.2 hours to enter mitosis while cells exposed to both BI 2536 and MLN8054 took 26.0 hours (Fig. 8D). While not statistically significant, most likely due to the sample size, this is consistent with prior observations showing that co-inhibition of Plk1 and Aurora A delays mitotic entry [46]. Thus, while inhibiting Aurora A further delays mitotic entry in Plk1-deficient cells that commit to mitosis, it does not increase the proportion that block in late G2.

### Combining BI 2536 with the Wee1 inhibitor MK-1775 alleviates the mitotic entry block

**Figure 9:**
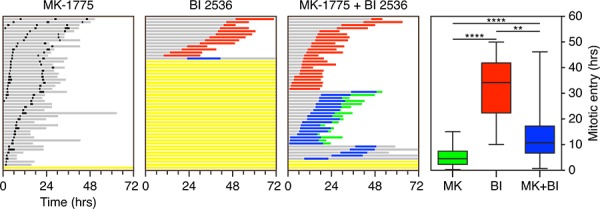
Co-inhibition of Plk1 and Wee1 Fate profiles of asynchronous RKO cells exposed to 100 nM MK-1775 and/or 250 nM BI 2536 for 72 hours. 50 cells were analyzed per condition. Note that zero hours represents when imaging started. Graph shows time from when imaging started to mitotic entry with box-and-whiskers showing the mean, interquartile ranges and full range. **p < 0.01, ****p < 0.0001.

Plk1 promotes mitotic entry by phosphorylating and activating Cdc25 phosphatases (reviewed in [46]). This in turn leads to dephosphorylation of Cdk1 on T14 and Y15, thereby alleviating Wee1-mediated inhibition of Cdk1/Cyclin B1. We reasoned that if the mitotic entry block induced by BI 2536 was due to inhibition of Plk1-mediated activation of Cdc25, then inhibition of Wee1 should alleviate the block and facilitate mitotic entry. To test this we treated cells with the Wee1 inhibitor MK-1775 [49]. RKO cells treated with 100 nM MK-1775 completed cell division apparently normally (Fig. 9). Consistent with observations described above, 250 nM BI 2536 blocked mitotic entry in 72% of cells and substantially delayed it in the remaining 28% (Fig. 9). Strikingly, upon simultaneous exposure to BI 2536 and MK-1775, 94% of cells entered mitosis and moreover, they did so sooner than those that entered in the presence of BI 2536 alone (13.9 versus 30.8 hours, Fig. 9). All the cells that entered mitosis underwent a prolonged arrest, followed by either death in mitosis or slippage, indicating that inhibiting Wee1 cannot bypass the need for Plk1 during mitosis. Interestingly, MK-1775 did not fully restore the timing of mitotic entry (13.9 versus 5.4 hours), possibly reflecting the activity of additional Plk1 targets responsible for Cdk1 inhibition such as Myt1 [46]. Nevertheless, these observations suggest that the mitotic entry block induced by BI 2536 is due to a failure to activate Cdc25.

## DISCUSSION

We show here a paradoxical relationship between Plk1 inhibitor concentration and the induction of cell death, whereby lower concentrations are more effective at inducing apoptosis. This paradoxical relationship has been observed before. When Raab and colleagues treated HeLa cells with BI 2536 at concentrations up to 100 nM, they observed potent apoptosis induction [49, 50]. At higher concentrations however, mitotic markers were less abundant and up to ~20% of cells survived. Our observations provide a simple explanation for this paradox: at higher concentrations, Plk1 inhibitors block mitotic entry thereby protecting cells from the apoptosis induction that typically follows a prolonged mitotic arrest. Six different Plk1 inhibitors, representing four different classes, all block mitotic entry, suggesting that this phenotype is unlikely due to a common off-target effect. Indeed, Plk1's ability to drive mitotic entry is a well-characterized function, conserved from yeast to man. In the fission yeast *S.pombe*, Cdk1/Cyclin B1 becomes active on the spindle pole in late G2 where it activates the Plo1 kinase [51]. This triggers a feedback loop that enhances Cdc25 and suppresses Wee1, in turn driving further activation of Cdk1/Cyclin B1 and mitotic entry. In human cells, Plk1 is activated on the centrosome many hours before mitotic entry [52]. This is mediated by Bora which induces a conformational change in Plk1, facilitating Aurora-A-mediated phosphorylation of Plk1's T-loop [46]. Via feedback on Cdc25 and Wee1, active Plk1 then helps drive Cdk1/Cyclin B1 activation and mitotic entry. In *C.elegans*, Cdk1 phosphorylates Bora/SPAT-1, enhancing its ability to bind Plk1 [53]. This latter observation closes the circle, giving rise to a model whereby low-level activation of Cdk1 triggers a Plk1-dependent feedback loop which then drives mitotic entry.

Our observations are consistent with this model. If the mitotic entry block we observe is due to penetrant inhibition of Plk1, and if Aurora A acts upstream of Plk1, then inhibiting Aurora A when Plk1 is fully blocked is predicted to have no effect. Indeed, at 100 nM BI 2536, ~50% of HeLa cells arrest in G2 and co-inhibition of Aurora A does not increase this. Of the ~50% cells that do enter mitosis, co-inhibiting Aurora A extends the mitotic entry delay, indicating that when Plk1 is not fully blocked, Aurora A does promote the feedback loop. A corollary is that when Plk1 is not fully blocked, co-inhibition of Aurora A does not shut down the network, indicating that Aurora is either not an essential component of the feedback network or that it was not fully inhibited in our experiments. Consistent with either possibility, 2 μM MLN8054 in isolation had no effect on mitotic entry timing.

The Cdk1 ➜Aurora A ➜Plk1 network exerts mitotic entry control at the post-translational level. However, Plk1 also promotes mitosis by regulating gene expression. Plk1 phosphorylates the forkhead transcription factor FoxM1 which in turn upregulates genes required for G2 progression and mitosis, including mitotic cyclins, the kinetochore protein Cenp-F and Plk1 itself [19, 54]. Thus, the Plk1-FoxM1 positive feedback maintains tight transcriptional control of mitotic entry. The ability of Plk1 inhibitors to either block cells in G2 or delay mitotic entry could therefore be a combination of inhibiting the transcriptional and/or post-translational controls. However, why some cells block in G2 and others only delay mitotic entry is unclear. Indeed, the variation we observe, both between cell lines and within the same line, is striking. In RKO, the proportion of cells that block in G2 increases with increasing inhibitor concentration, approaching >90%. However, in HeLa cells, the proportion that arrests in G2 plateaus at ~50%. Thus, while the extent of the G2 block is titrateable, the plateau differs from line to line, indicating interline heterogeneity. While this may be due to genetic differences between the lines, we also observed intraline variation; specifically, daughter cells subjected to identical environmental conditions often behaved differently upon exposure to Plk1 inhibitors. This intraline variation appears to be another example of non-genetic heterogeneity [26], suggesting that the mitotic entry feedback networks described above are differentially sensitive to Plk1 inhibition. Why this is the case is unclear, but interestingly the rate of Plk1-dependent recovery from DNA damage is highly variable [37], further supporting the notion that the mitotic entry feedback networks are subject to stochastic variation.

Our observations also suggest that different levels of Plk1 activity are required to drive progress through different stages of the cell cycle; while a relatively low level is required to promote mitotic entry, a higher level is required to assemble a bipolar spindle. Because five out of the six Plk1 inhibitors we analyzed are not fully reversible, this gives rise to an interesting phenomenon following Plk1 inhibitor washout; specifically, the mitotic entry block is alleviated but cells then arrest in mitosis due to an inability to assemble a functional spindle, in turn leading to apoptosis, either in mitosis or following slippage. This observation has important clinical implications, because it suggests that Plk1 inhibitors may be able induce apoptosis even when cells do not pass through mitosis during the time period where the drug reaches maximal plasma concentration. If this is the case, then the less-reversible inhibitors may be more effective. By the same token however, this may also increase the toxicity towards dividing non-tumor cells. Of the six Plk1 inhibitors we analyzed, CYC140844 stands out because it does appear to be fully reversible. In contrast with the others, while CYC140844 induces a potent G2 arrest, following washout the population was indistinguishable from the control. CYC140844 could therefore be a useful cell synchronization tool. Indeed, when we treated an asynchronous population of RKO cells with 500 nM CYC140844 for eight hours, ~80% of the cells entered mitosis within four hours of washout. By contrast, it took over 9 hours for a similar number of control cells to enter (Fig. 7B). Optimization of this approach, and for example combining it with a prior G1/S block-and-release, could result in a highly effective synchronization regime.

## EXPERIMENTAL PROCEDURES

### Cell lines and small molecules

TA-HeLa, DLD-1, RKO and RPE-1 cells expressing GFP-tagged histone H2B were as described [26]. All cell lines were cultured in DMEM plus 10% fetal calf serum (LifeTechnologies), 2 mM glutamine, 100 U/ml penicillin and 100 U/ml streptomycin (Lonza). All lines were grown at 37°C in a humidified 5% CO_2_ incubator. Small molecule inhibitors dissolved in DMSO were as follows: BI 2536 (Boehringer Ingelheim); monastrol (Sigma); CYC140844 (Cyclacel Ltd. (Cyclacel Ltd.)); BI 6727, MLN0905 (Axon MedChem), RO3280, MK-1775 (Selleck Chemicals); TAK-960 (R&D Systems); MLN8054 (Millennium Pharmaceuticals). Thymidine (Sigma), dissolved in water and filter sterilized was used at 2 mM.

### Immunofluorescence

To determine the cell cycle phase of interphase-arrested cells, 1.4 × 10^5^ cells were seeded onto 19 mm UV-sterilized glass coverslips, cultured in the presence of 100 nM BI 2536, then fixed in 1% formaldehyde in PBS for five minutes (Cenp-F) or in ice-cold 50:50 methanol:acetone for 10 min at −20°C (Cyclin B1). Following washes in PBS plus 0.1% Triton X-100 (PBST), formaldehyde-fixed cells were quenched in glycine pH 8.5 for 20 min then incubated for 30 minutes with combinations of primary antibodies diluted in PBS-glycine (CENP-F) or PBST (Cyclin B1 coverslips) as follows: sheep anti-Cenp-F SCF1.1 1:1000 [55]; and mouse anti-Cyclin B1 (Upstate) 1:1000. Following washes in PBST, the cells were incubated with appropriate combinations of the following secondary antibodies, all diluted 1:500 in PBST: Cy3 conjugated donkey anti-mouse and Cy3 conjugated donkey anti-sheep (both from Jackson Immunoreseach). Following washes in PBST, the cells were stained with Hoechst 33258 at 1 μg/ml in PBST and mounted in 90% glycerol, 20 mM Tris-HCl pH 8. Cells were viewed on an Axioskop 2 microscope (Zeiss), images captured using a CoolSnap HQ CCD camera (Photometrics) driven by Metamorph software (Molecular Devices). To determine spindle morphology, cells were seeded on coverslips as described above and inhibitors added at the required concentration for 3 hours. To visualize microtubules, cells were pre-extracted in PEM-T buffer (100 mM PIPES, 1 mM MgCl_2_, 0.1 mM CaCl_2_, 0.1% Triton X-100) for 90 s. Cells were then fixed in 4% formaldehyde in PEM-T for 10 minutes, quenched in PBS-glycine for 20 minutes and stained with the following primary antibodies: rabbit anti-phospho-histone H3 (Ser10) 1:500 (Millipore) and TAT-1 mouse anti-tubulin 1:200 (Woods et al 1992). Secondary antibodies (1:500) used were: Cy2 conjugated donkey anti-rabbit and Cy3 conjugated donkey anti-mouse (both from Jackson Immunoreseach).

### Time-lapse imaging

For time-lapse microscopy, cells were seeded in 96-well μClear plates (Greiner) at 1.3 × 10^4^ cells per well in a volume of 100 μl. Synchronization was performed by adding thymidine at a final concentration of 2 mM four hours post-seeding then removed 16 hours later by washing three times with PBS followed by adding fresh media. Four hours after thymidine washout, additional media containing inhibitor(s) was added and imaging then started one hour later. Fluorescence imaging was then performed using a Pathway Bioimager 855 (BD Biosciences), BD Attovision software and a 20x/0.30 UPlan FLN objective. 96-well plates were housed in a customized chamber to maintain a constant temperature of 37°C and a humidified 5% CO_2_ atmosphere. Images were collected every 5 min as a 2 × 2 montage using an Orca ER camera (Hamamatsu) with a 0.1 s exposure time. For analysis of daughter pairs, imaging was performed for 24 hours in drug-free media prior to addition of fresh media containing inhibitor. Image sequences were then viewed using NIH ImageJ software. Cells were tracked manually and behavior determined by visual inspection of GFP histone H2B morphology. Timing data was analyzed and sorted using Microsoft Excel then cell fate profiles and other graphs created using GraphPad Prism.

### Flow cytometry

To determine the DNA content and mitotic index by flow cytometry, cells were cultured in 10 cm dishes in the presence of the appropriate inhibitors. Culture media was then collected and the remaining cells harvested by trypsinisation. Cells were then pelleted and washed in PBS before finally being resuspended in 150 μl PBS plus 350 μl 100% ice-cold ethanol. Cells were then stored at −20°C for at least 16 hr. Fixed cells were washed twice in PBS and incubated with rabbit anti-phospho-histone H3 (Ser10)(Millipore) diluted 1:1000 in PBS for 1 hour at 4°C. Following a PBS wash, cells were then incubated with a FITC-conjugated anti-rabbit antibody (Jackson Immunoresearch) for one hour at 4°C. Cells were again washed in PBS and resuspended in 500 μl PBS containing 40 μg/ml propidium iodide, 50 μg/ml RNase A. Samples were then incubated in the dark at room temperature for 30 min before being analyzed on a Cyan ADP flow cytometer using Summit analysis software (Dako).

## SUPPLEMENTARY FIGURE


