# Supplementary material for: Mitotic entry: Non-genetic heterogeneity exposes the requirement for Plk1
Source: Oncotarget. 2015 Oct 13;6(34):36472–88. doi: 10.18632/oncotarget.5507 (PMC4742190; doi:10.18632/oncotarget.5507)
Supplement: Supplementary file 1 [file oncotarget-06-36472-s001.pdf]

SUPPLEMENTARY FIGURE

R group	Modifications
R1	Alkyl, aryl, benzyl
R2	Alkyl variants
R3	Alkyl variants
X	O, S
R4	H, Me, Et and ^{i}Pr
R5	OMe, OCF_3, OEt, H, Hal, OH
R6	CO_2H, OH, OMe, Amides

Supplementary Figure S1: R group moieties for CYC140844.
